# Dactylogyrids (Platyhelminthes, Monogenea) from the gill lamellae of doradids (Siluriformes) with description of five new species of *Cosmetocleithrum* and new geographical distribution for known species from the Neotropical Region, Brazil[Fn FN1]

**DOI:** 10.1051/parasite/2023054

**Published:** 2023-12-05

**Authors:** Augusto Leandro de Sousa Silva, Yuri Costa de Meneses, Williane Maria de Oliveira Martins, Simone Chinicz Cohen, Andréa Pereira da Costa, Marcia Cristina Nascimento Justo

**Affiliations:** 1 Programa de Pós-graduação em Ciência Animal, Laboratório de Multiusuários em Pesquisa da Pós-graduação (LAMP) – Universidade Estadual do Maranhão (UEMA). Cidade Universitária Paulo VI Avenida Lourenço Vieira da Silva, 1000 São Luís Maranhão, MA Brazil; 2 Laboratório de Helmintos Parasitos de Peixes, Instituto Oswaldo Cruz, FIOCRUZ Av. Brasil, 4365 Rio de Janeiro RJ 21045-900 Brazil; 3 Laboratório de Biologia Geral do Instituto Federal de Acre (IFAC), Campus Cruzeiro do Sul Estrada da Apadec no. 1192, Bairro Nova Olinda CEP: 69980-000 Cruzeiro do Sul Acre Brazil; 4 Laboratorio de Parasitologia e Doenças Parasitárias dos Animais – LPDP, UEMA, Cidade Universitária Paulo VI Avenida Lourenço Vieira da Silva, 1000 São Luís Maranhão, MA Brazil

**Keywords:** *Cosmetocleithrum*, Dactylogyridae, Monogeneans, Doradidae, South America

## Abstract

Five new species of *Cosmetocleithrum* were described parasitizing the gill filaments of neotropical doradid fishes. *Cosmetocleithrum undulatum* n. sp., *Cosmetocleithrum brachylecis* n. sp. and *Cosmetocleithrum ludovicense* n. sp. are described from *Platydoras brachylecis* from a market-place of São Luís, State of Maranhão, Brazil. *Cosmetocleithrum sacciforme* n. sp. and *Cosmetocleithrum basicomplexum* n. sp. are described from *Oxydoras niger* from Juruá River, State of Acre, Brazil. *Cosmetocleithrum undulatum* and *Cosmetocleithrum brachylecis* resemble *Cosmetocleithrum falsunilatum* Feronato, Razzolini, Morey & Boeger, 2022 mainly by the unique male copulatory organ (MCO) morphology but differ from these and all congeneric species mainly by the morphology of the MCO, accessory piece and hooks pairs. *Cosmetocleithrum ludovicense* is closer to *Cosmetocleithrum confusus* Kritsky, Thatcher & Boeger, 1986 and to *Cosmetocleithrum akuanduba* Soares, Santos Neto & Domingues, 2018 but differs from those mainly by the morphology of the accessory piece. *Cosmetocleithrum sacciforme* differs from all congeneric species mainly by the morphology of the accessory piece formed by a single plate of saccular appearance. *Cosmetocleithrum basicomplexum* also shares morphological characters with *Cosmetocleithrum gigas* Morey, Cachique & Babilonia, 2019 considering the size of the body and shape of the anchors, but differs mainly in the morphology of the bars and hooks. Besides the new species, new data are presented for *Cosmetocleithrum leandroi* Soares, Neto & Domingues, 2018*, C. akuanduba* and *C. confusus* regarding morphological characteristics and biogeography.

## Abstract (fr)

Cinq nouvelles espèces de *Cosmetocleithrum* sont décrites, parasitant les filaments branchiaux de poissons Doradidae néotropicaux. *Cosmetocleithrum undulatum* n. sp., *Cosmetocleithrum brachylecis* n. sp. et *Cosmetocleithrum ludovicense* n. sp. sont décrits de *Platydoras brachylecis* provenant d’un marché de São Luís, État du Maranhão, Brésil. *Cosmetocleithrum sacciforme* n. sp. et *Cosmetocleithrum basicomplexum* n. sp. sont décrits d’*Oxydoras niger* de la rivière Juruá, État d’Acre, Brésil. *Cosmetocleithrum undulatum* et *Cosmetocleithrum brachylecis* ressemblent à *Cosmetocleithrum falsunilatum* Feronato, Razzolini, Morey & Boeger, 2022 principalement par la morphologie unique de l’organe copulateur mâle (OCM), mais diffèrent de ces espèces et de toutes les espèces congénères principalement par la morphologie de l’OCM, de la pièce accessoire et des paires de crochets. *Cosmetocleithrum ludovicense* est proche de *Cosmetocleithrum confusus* Kritsky, Thatcher & Boeger, 1986 et de *Cosmetocleithrum akuanduba* Soares, Santos Neto & Domingues, 2018 mais en diffère principalement par la morphologie de la pièce accessoire. *Cosmetocleithrum sacciforme* se distingue de toutes les espèces congénères principalement par la morphologie de la pièce accessoire formée d’une seule plaque d’aspect sacculaire. *Cosmetocleithrum basicomplexum* partage également des caractères morphologiques avec *Cosmetocleithrum gigas* Morey, Cachique & Babilonia, 2019 compte tenu de la taille du corps et de la forme des anchors, mais en diffère principalement par la morphologie des barres et des crochets. Outre les nouvelles espèces, de nouvelles données sont présentées pour *Cosmetocleithrum leandroi* Soares, Neto & Domingues, 2018, *C. akuanduba* et *C. confusus* concernant les caractéristiques morphologiques et la biogéographie.

## Introduction

The neotropics, spanning from central Mexico to the southern limit of South America, has the most diverse group of fishes on the planet [14]. This region harbors the greatest diversity of freshwater fish with approximately 6,000 known species and estimates of 9,000 species [18]. Of the total known species, the Characiformes, Siluriformes, and Gymnotiformes account for approximately 77% of the known species [2]. Thus, South America harbors the most diverse fauna of continental freshwater fish in the world with approximately 5,750 known species [3]. Siluriformes, collectively known as catfish, stand out as the largest and most diverse order of freshwater fish, and constitute one of the most important components of the Neotropical fauna, with more than 3,800 described species [6]. Among all the families of this order, Doradidae stand out as one of the most diverse and representative families among the Neotropical Siluriformes, with more than 90 valid species [6]. Doradidae are a monophyletic group endemic to freshwaters of South America on both sides of the Andes Mountains [19].

Siluriformes host a remarkably rich and diverse fauna of gill monogeneans, and these host-parasite systems are an attractive model for phylogenetic studies in the Neotropics [4, 12]. The dactylogyrid *Cosmetocleithrum* Kritsky, Thatcher & Boeger, 1986 is one of the most species-rich groups of monogenoids reported from siluriform fishes and was proposed to accommodate species of Dactylogyridae that parasitize *Oxydoras niger* (Valenciennes) and *Pterodoras granulosus* (Valenciennes) in the Amazon River basin [9]. This genus shows high specificity to catfishes within Doradidae and Auchenipteridae [11]. Currently, 23 species of *Cosmetocleithrum* are recognized as parasites of the gills of neotropical Siluriformes, among which 15 species recorded in doradids hosts, eight in Auchenipteridae, one parasitizing Pimelodidae, and one in Loricariidae. All species of *Cosmetocleithrum* have been described from hosts of members of a single family [1, 4, 5, 15, 22, 23, 24], except for *Cosmetocleithrum bulbocirrus* Kritsky, Thatcher & Boeger, 1986, reported from species of three different siluriform families and from *Hoplias malabaricus* (Bloch), a characiform fish [7]. However, considering that only one specimen was found in the latter host, this record needs to be confirmed.

During research on monogeneans of siluriform fishes in northern and northeastern Brazil, five new species of *Cosmetocleithrum* were found and are described herein. Moreover, new data are presented to *Cosmetocleithrum leandroi* Soares, Neto & Domingues, 2018*, Cosmetocleithrum akuanduba* Soares, Santos Neto & Domingues, 2018 and *Cosmetocleithrum confusus* Kritsky, Thatcher & Boeger, 1986 regarding morphological characteristics and biogeographical data, thus expanding the knowledge of these species.

## Material and methods

The studies were carried out between 2019 and 2023 on different doradid species collected from two distinct localities in Brazil. Specimens of *Oxydoras niger* were captured with gill nets and hook and line from Juruá River, Acre, Brazil (7°40′34.1″S, 72°39′39.5″W) and those from *Platydoras brachylecis* and *Hassar affinis* were obtained from street markets located on the island of São Luís, State of Maranhão (2°34′18.0″S 44°11′49.9″W).

The gills of each specimen were removed and placed in vials containing hot water (*c.* 65 °C) in order to relax the parasites, and they were then shaken to detach the parasites from the gill filaments. Subsequently, absolute ethanol was added to reach a concentration of 70%. The vials were then sent to “Laboratório de Helmintos Parasitos de Peixes, Instituto Oswaldo Cruz, FIOCRUZ”, where the gills were analyzed, and the parasites identified. Monogeneans were picked using a stereoscopic microscope for subsequent morphological studies. Some specimens were mounted in Hoyer’s medium for study of the sclerotized parts; others were stained with Gomori’s trichrome for study of the internal organs of the parasite [8]. Measurements are presented in micrometers; range values are followed by mean and number of structures measured in parentheses. Dimensions of organs and other structures represent the greatest distance; lengths of curved or bent structures (anchors, bars, and accessory piece) represent the straight-line distances between extreme ends [9], except for copulatory complexes that were measured using ImageJ [17]. The specimens were studied, photographed, and drawn using an Olympus BX 41 microscope with phase contrast and Zeiss Axioskop 2 Plus microscope with differential interference contrast (DIC), both equipped with a camera lucida. Holotypes, paratypes, and vouchers of each species were deposited in the “Coleção Helmintológica do Instituto Oswaldo Cruz - CHIOC”, from “Fundação Oswaldo Cruz - FIOCRUZ”.

## Results

Class Monogenea Bychowsky, 1937

Subclass Monopisthocotylea Odhner, 1912

Order Dactylogyridea Bychowsky, 1937

Dactylogyridae Bychowsky, 1933

*Cosmetocleithrum* Kritsky, Thatcher & Boeger, 1986

## *Cosmetocleithrum undulatum* n. sp. (Fig. 1)

urn:lsid:zoobank.org:act:D915B062-6777-4D6F-8024-C5CF6904F062

**Figure 1:**
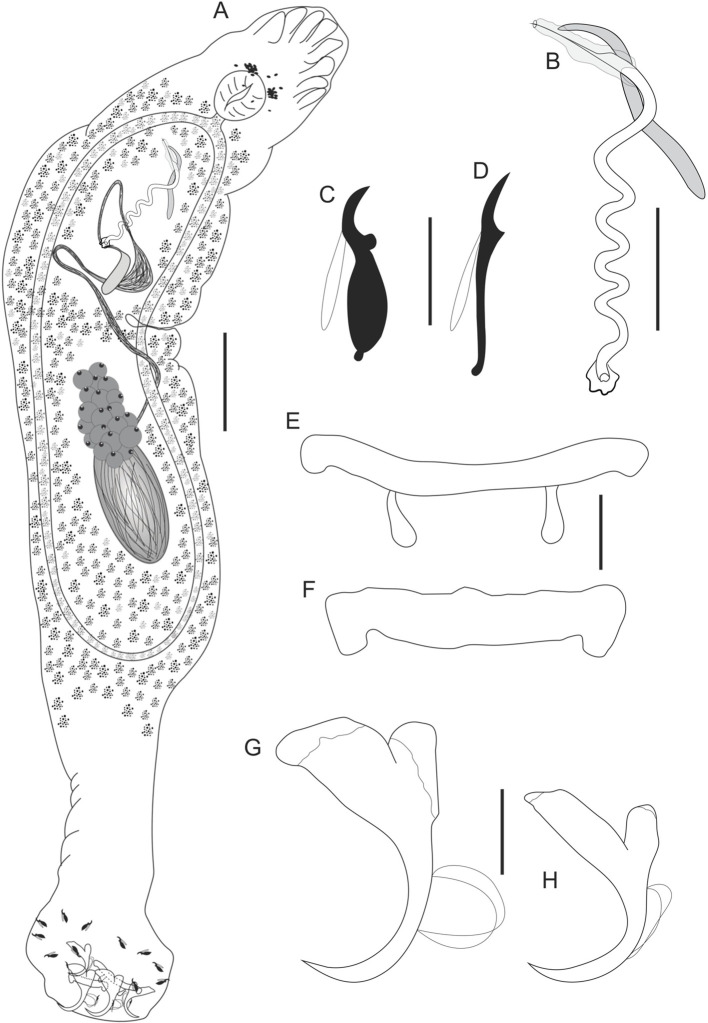
*Cosmetocleithrum undulatum* n. sp. from *Platydoras brachylecis*. (A) Total ventral view (composite); (B) Copulatory complex, ventral view; (C) Hook pairs 1–4, 6, 7; (D) Hook pair 5; (E) Dorsal bar; (F) Ventral bar; (G) Ventral anchor; (H) Dorsal anchor. Scale bars: A, 100 μm, B, C, 10 μm, D, 50 μm, E, F, 15 μm, G, H, 20 μm.

Type-host: *Platydoras brachylecis* Piorski, Garavello, Arce H & Sabaj Pérez, 2008 (Siluriformes, Doradidae).

Site in host: Gill lamellae.

Type-locality: Market-place of the Cidade Operária, São Luís, State of Maranhão, Brazil (2°34′18.0″S 44°11′49.9″W).

Parasitological indexes: Total number of hosts: 3; number of infected hosts: 3; total number of parasites: 312.

Type-material: Holotype CHIOC 40257 a; Paratypes CHIOC 40257 b–h; 40258 a–g.

Etymology: The specific name is derived from Latin (*undulatum* = wavy) and refers to the shape of the male copulatory organ.

### Description

Based on 21 specimens: 14 mounted in Hoyer’s medium and 7 stained in Gomori’s trichrome. Body fusiform, elongated, comprising cephalic region, trunk, peduncle, and haptor. Tegument thin, smooth. Body, including haptor, 1069 (780–1380; *n* = 14) long by 228 (110–320; *n* = 14) wide at the level of germarium. Cephalic lobes poorly developed. Four pairs of head organs. Cephalic glands indistinct. Eyes absent. Accessory granules scattered in the pharyngeal region, sometimes gathered, resembling eyes. Mouth subterminal. Pharynx spherical 52 (32–73; *n* = 7) in diameter. Esophagus short. Two intestinal ceca confluent posteriorly to testis, lacking diverticula. Gonads intercecal, tandem. Germarium 97 (83–125; *n* = 4) long by 21 (14–36; *n* = 4) wide. Vagina simple, non-sclerotized; vaginal aperture sinistroventral. Seminal receptacle pyriform. Mehlis’ gland, uterus, oviduct, and ootype not observed. Vitellaria extends throughout the trunk, except in areas of other reproductive organs. Testis postgermarial, 49 (32–60; *n* = 3) long by 20 (15–30; *n* = 3) wide. Vas deferens looping left intestinal cecum; seminal vesicle a dilatation of vas deferens. Prostatic reservoir elongated. Copulatory complex comprising male copulatory organ (MCO) and accessory piece. MCO formed by a wavy tube and sclerotized walls that abruptly tapers at the tip; a thin, and delicate veil-shaped layer covers the entire tip of the cirrus and involves the accessory piece in its distal portion, 220 (200–249; *n* = 14) in total length. Accessory piece straight, non-articulated to the base of the MCO, 98 (86–116; *n* = 14) long. Peduncle conspicuous, long. Haptor globose, with dorsal and ventral anchor/bar complexes, 177 (110–220; *n* = 14) wide. Ventral bar straight with enlarged ends projecting posteriorly, 57 (49–72; *n* = 14) wide. Dorsal bar more straight with two submedian projections, 69 (57–85; *n* = 14) wide. Ventral and dorsal anchors dissimilar in size and shape. Ventral anchor with well-developed, square-shaped superficial root and rounded deep root, curved shaft and point not passing from the level of the tip of superficial root, outer 59 (55–61; *n* = 14); inner 50 (47–54; *n* = 14); base 33 (29–40; *n* = 14). Dorsal anchor with well-developed rectangular superficial root and well-developed and rounded deep root, evenly curved shaft and point not passing from the level of the tip of superficial root, outer 41 (39–43; *n* = 14); inner 37 (35–43; *n* = 14); base 26 (22–30; *n* = 14). Hooks with ancyrocephaline distribution. Hooks dissimilar, pair 5 with a delicate point, inconspicuous thumb, slender straight shank with point slightly recurved, filamentous hook (FH) loop 3/4 shank length, 18 (17–19; *n* = 13); hook pairs 1–4, 6 and 7 with shaft powerfully robust through its length, ending in a small rounded portion at the end; thumb short, rounded; filamentous hook (FH) loop about shank length, 16 (15–18; *n* = 78).

### Remarks

*Cosmetocleithrum undulatum* n. sp. differs from all congeneric species mainly in terms of the morphology of the MCO, while resembling *C. falsunilatum* Feronato, Razzolini, Morey & Boeger, 2022 with a unique MCO morphology, very similar to the species of *Unilatus* Mizelle & Kritsky, 1967, that are parasites of loracariids. However, *Cosmetocleithrum undulatum* n. sp. differs from *C. falsunilatum* regarding the shape of the MCO, which in *C. falsunilatum* is rolled up into a corkscrew shaped, while in *Cosmetocleithrum undulatum* n. sp. the MCO is an undulating tube with a thin and delicate layer that covers the entire tip of the MCO. Furthermore, the accessory piece of *C. falsunilatum* is subdivided into two regions that reach close to the base of the MCO, while in the new species, the accessory piece is straight, reaching just less than half of the length of the MCO. The hook pairs 1–4, 6, 7 of the new species are unique among the *Cosmetocleithrum* species, with a shaft that is strongly robust throughout its length, and with a small constricted portion ending with a small rounded portion at the end.

## *Cosmetocleithrum brachylecis* n. sp. (Fig. 2)

urn:lsid:zoobank.org:act:D915B062-6777-4D6F-8024-C5CF6904F062

**Figure 2:**
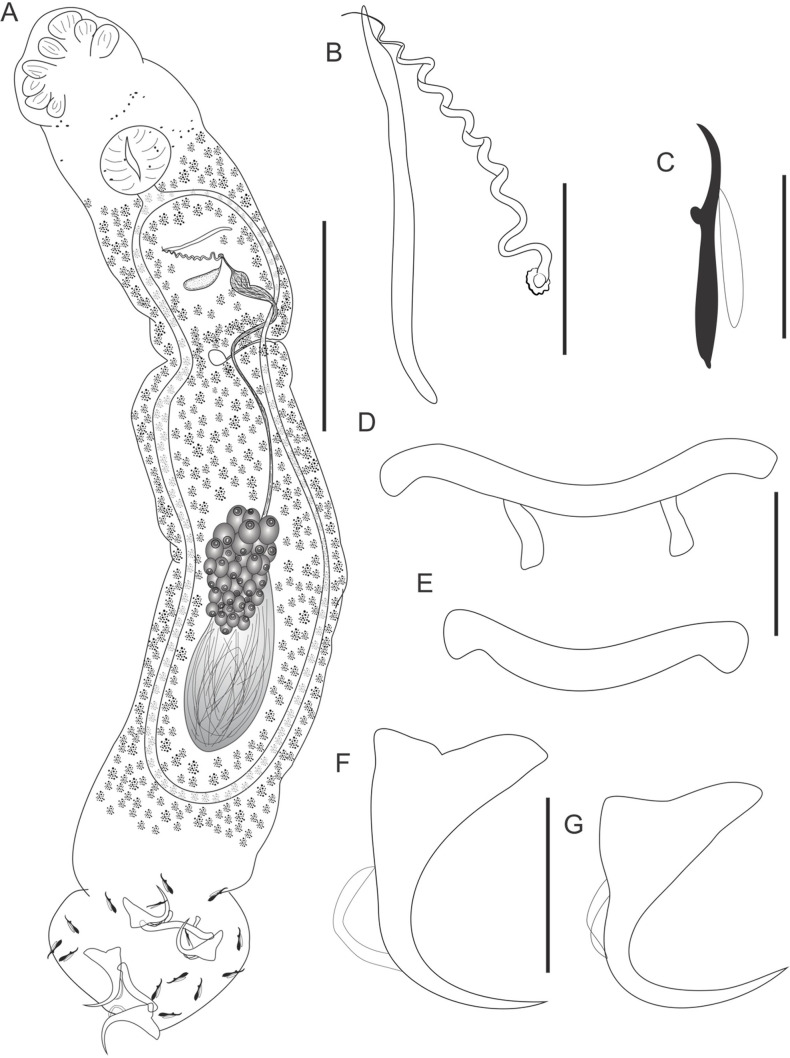
*Cosmetocleithrum brachylecis* n. sp. from *Platydoras brachylecis*. (A) Total, ventral view (composite); (B) Copulatory complex, ventral view; (C) Hook; (D) Dorsal bar; (E) Ventral bar; (F) Ventral anchor; (G) Dorsal anchor. Scale bars: A, 100 μm, B, D–G, 20 μm, C, 10 μm.

Type-host: *Platydoras brachylecis* Piorski, Garavello, Arce H & Sabaj Pérez, 2008 (Siluriformes, Doradidae).

Site in host: Gill lamellae.

Type-locality: Market-place of the Cidade Operária, São Luís, State of Maranhão, Brazil (2°34′18.0″S 44°11′49.9″W).

Parasitological indexes: Total number of hosts: 3; number of infected hosts: 3; total number of parasites: 38.

Type-material: Holotype CHIOC 40247 a; Paratypes CHIOC 40247 b–m; 40248.

Etymology: The specific name is derived from the name of its host, *Platydoras brachylecis.*

### Description

Based on 16 specimens mounted in Hoyer’s medium and 3 stained in Gomori’s trichrome. Body fusiform, elongated, comprising cephalic region, trunk, peduncle, and haptor. Body, including haptor, 528 (360–660; *n* = 13) long by 116 (60–160; *n* = 13) wide at the germarium level. Tegument thin, smooth. Cephalic lobes poorly developed. Four pairs of head organs. Cephalic glands indistinct. Eyes absent. Accessory granules scattered in the cephalic and pharyngeal region. Mouth subterminal. Pharynx spherical, muscular 34 (27–43; *n* = 9) in diameter. Esophagus short. Two intestinal ceca confluent just posteriorly to gonads, lacking diverticula. Gonads tandem, germarium pre-testicular. Germarium 110 (72–157; *n* = 4) long by 23 (17–23; *n* = 4) wide. Vagina simple, non-sclerotized; vaginal aperture sinistroventral. Seminal receptacle rounded. Mehlis’ gland, uterus, oviduct and ootype not observed. Vitelline follicles dense, dispersed throughout trunk but absent in region of reproductive organs and MCO. Testis posterior to germarium 27 (22–32; *n* = 4) long by 27 (22–35; *n* = 4) wide. Vas deferens looping left intestinal cecum; seminal vesicle a dilatation of vas deferens. Prostatic reservoir anterior to seminal vesicle. Copulatory complex comprising male copulatory organ (MCO) and accessory piece. MCO composed as a spiral tube with sclerotized walls, with counter-clockwise orientation, 68 (49–82; *n* = 13) in total length. Accessory piece straight, elongated, exceeding the base of the cirrus, non-articulated to the base, 45 (36–52; *n* = 13) long. Peduncle elongate. Haptor globose, almost the same width as the body 104 (60–130; *n* = 13) wide. Ventral bar slightly recurved with enlarged ends, 38 (24–43; *n* = 13) wide. Dorsal bar robust, broadly u-shaped, with two submedian projections, 46 (25–55; *n* = 13) wide. Ventral and dorsal anchors dissimilar in size and similar in shape, robust superficial and deep roots; deep root straight; superficial root pointed; evenly curved shaft, and short point. Ventral anchor outer 37 (35–39; *n* = 13); inner 32 (27–43; *n* = 13); base 23 (19–26; *n* = 13). Dorsal anchor outer 29 (26–32; *n* = 13); inner 22 (18–25; *n* = 13); base 19 (16–20; *n* = 13). Hooks with ancyrocephaline distribution. Hooks similar in shape and size; point and shaft delicate, erect thumb, shank expanded, tapering abruptly proximally in a point, 15 (13–16; *n* = 70); FH loop about 3/4 shank length.

### Remarks

*Cosmetocleithrum brachylecis* n. sp. resembles *C. falsunilatum* and *C. undulatum* n. sp. regarding the shape of the MCO, which is similar to the unique feature of *Unilatus*. While the MCO in *C. falsunilatum* has a cork-screw-like shape, the MCO in the new species is formed by a spiral tube with sclerotized walls. The new species differs from *C. undulatum* n. sp. regarding the lengths of the MCO and accessory piece: in the new species, the accessory piece is straight and elongated, such that it goes beyond the base of the cirrus, while in *C. undulatum* it is also straight, but only reaches just less than half of the length of the MCO. The new species also differs from all congeneric species in terms of the morphology of the hooks, which are distally slender, expanded in the middle third, and taper abruptly proximally.

## *Cosmetocleithrum ludovicense* n. sp. (Fig. 3)

urn:lsid:zoobank.org:act:2733EAE7-3CF5-4BBD-804A-12852B7348E7

**Figure 3:**
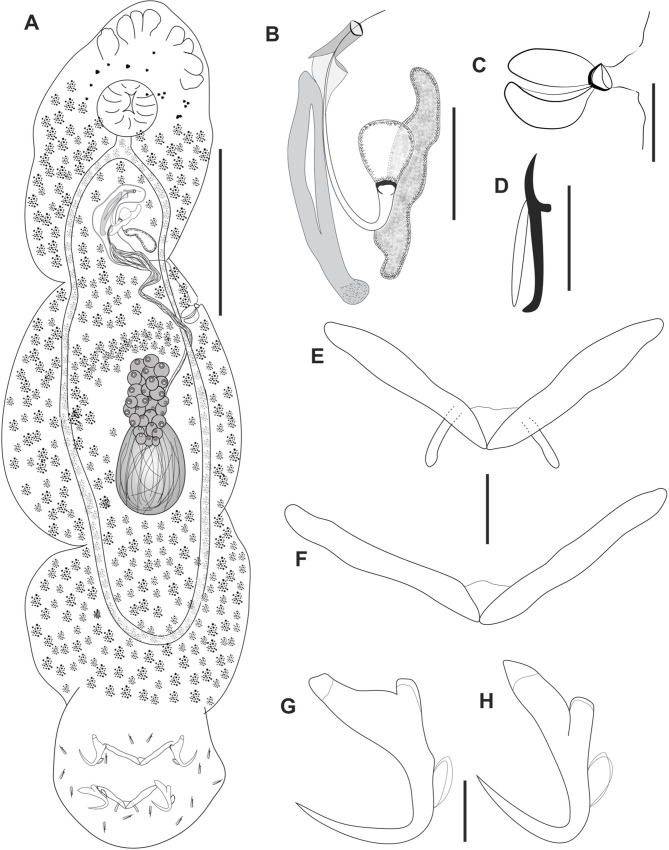
*Cosmetocleithrum ludovicense* n. sp. from *Platydoras brachylecis*. (A) Total, ventral view (composite); (B) Copulatory complex, ventral view; (C) Vagina; (D) Hook; (E) Dorsal bar; (F) Ventral bar; (G) Ventral anchor; (H) Dorsal anchor. Scale bars: A, 100 μm, B, C, 20 μm, D–H, 10 μm.

Type-host: *Platydoras brachylecis* Piorski, Garavello, Arce H & Sabaj Pérez, 2008 (Siluriformes, Doradidae).

Site in host: Gill lamellae.

Type-locality: Marketplace of the Cidade Operária, São Luís, State of Maranhão, Brazil (2°34′18.0″S 44°11′49.9″W).

Parasitological indexes: Total number of hosts: 3; number of infected hosts: 3; total number of parasites: 434.

Type-material: Holotype CHIOC 40251; Paratypes CHIOC 40252 a–c; 40253 a–q.

Etymology: The specific name is in honor of people born on São Luís Island, state of Maranhão, Brazil.

### Description

Based on 25 specimens: 20 mounted in Hoyer’s medium and 5 stained in Gomori’s trichrome. Body fusiform, elongated, comprising cephalic region, trunk, peduncle and haptor. Body, including haptor, 510 (310–630; *n* = 20) long by 119 (50–170; *n* = 20) width at level of germarium. Tegument thin, smooth. Cephalic lobes poorly developed. Four bilateral pairs of head organs. Cephalic glands indistinct. Eyes absent. Accessory granules sometimes scattered in the pharyngeal region. Mouth subterminal. Pharynx spherical, weakly muscular 36 (33–50; *n* = 12) in diameter. Esophagus short. Two intestinal ceca confluent just posteriorly to testis, lacking diverticula. Gonads intercecal, tandem. Germarium 35 and 63; long by 22 and 40 wide. Vagina well-developed, formed by two chambers with heavily sclerotized walls that come together to form an opening; vaginal canal very long, and convoluted, making some loops in the direction of MCO. Seminal receptacle small, slightly rounded. Mehlis’ gland, uterus, oviduct and ootype not observed. Vitellaria extends throughout the trunk, except in areas of other reproductive organs. Testis postgermarial 31 (21–38; *n* = 4) long by 21 (15–30; *n* = 4) wide. Vas deferens looping left intestinal cecum; seminal vesicle a dilatation of vas deferens. Prostatic reservoir pyriform. Copulatory complex comprising MCO and accessory piece. MCO a sclerotized tube, inverted J-shaped, 57 (47–67; *n* = 23) in total length; distal part formed by a small and delicate tube, presenting a cap through which its tip penetrates. MCO base irregularly expanded, with an oval sclerotized brim associated with the base and a conspicuous rod-shape flange. Accessory piece straight and robust, with a hollow structure and sclerotized walls, 40 (33–44; *n* = 23) long, non-articulated to the base of the MCO. Peduncle short. Haptor, subhexagonal, with dorsal, ventral anchor/bar complex, 94 (45–130; *n* = 15) wide. Hooks with ancyrocephaline distribution. Anchors similar in shape and size, with well-developed roots. Ventral anchor with elongate and truncated superficial root and short deep root, straight shaft and long point; point passing tip of superficial root, outer 23 (21–25; *n* = 23); inner 16 (12–20; *n* = 23); base 16 (10–20; *n* = 23). Dorsal anchor with elongated and pointed superficial root and short deep root, straight shaft and long point; point passing tip of superficial root, outer 23 (21–25; *n* = 23); inner 15 (13–18; *n* = 23); base 15 (13–18; *n* = 23). Ventral and dorsal bar open V-shaped, with a fracture of the medium region. Ventral bar 48 (20–65; *n* = 23) wide. Dorsal bar with two submedial projections, 43 (20–59; *n* = 24) wide. Hooks similar in size and shape, all of them comprising a curved base, slender shank, rounded and protruding thumb, curved shaft and short point 14 (12–15; *n* = 77) long; FH loop almost the total shank length.

### Remarks

*Cosmetocleithrum ludovicense* n. sp. is closely related to *Cosmetocleithrum confusus* and to *Cosmetocleithrum sobrinus* Kritsky, Thatcher & Boeger, 1986 regarding the enlarged base of the MCO. However, it differs from these species in terms of the shape of the accessory piece, which in *C. confusus* is a hollow structure with sclerotized walls and truncated end, and in *C. sobrinus* is large, globose and apparently hollow, while in the new species, the MCO is formed by a thin hollow tube of inverted J-shaped, with sclerotized walls. The new species is also closer to *Cosmetocleithrum akuanduba*, in which the MCO has a tubular coiled shaft that frequently appears to have an inverted J-shaped but differs from the latter in terms of its sclerotized and bulbous base in the latter.

## *Cosmetocleithrum sacciforme* n. sp. (Fig. 4)

urn:lsid:zoobank.org:act:E6C1543F-3AA1-438A-8AF7-12CDE13180F5

**Figure 4:**
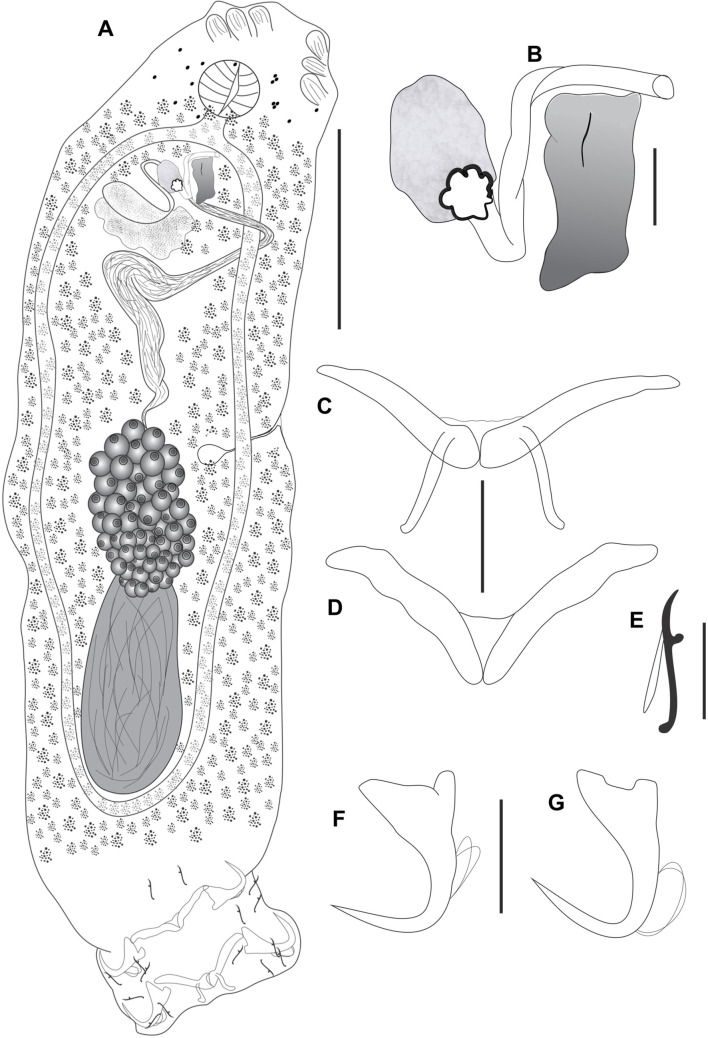
*Cosmetocleithrum sacciforme* n. sp. from *Oxydoras niger.* (A) Total, ventral view (composite); (B) Copulatory complex, ventral view; (C) Dorsal bar; (D) Ventral bar; (E) Hook; (F) Ventral anchor; (G) Dorsal anchor. Scale bars: A, 100 μm, B, E, 10 μm, C, D, F, G, 20 μm.

Type-host: *Oxydoras niger* (Valenciennes, 1821) (Siluriformes, Doradidae).

Site in host: Gill lamellae.

Type-locality: Juruá River, Acre, Brazil (7°40′34.1″S, 72°39′39.5″W).

Parasitological indexes: Total number of hosts: 7; number of infected hosts: 2; total number of parasites: 7.

Specimens deposited: Holotype CHIOC 40254; Paratypes CHIOC 40255 a–b; 40256 a–d.

Etymology: The specific name is from Latin (*saccus* = sac; *formis* = shape of) and refers to the shape of accessory piece, which resembles a sac-like structure.

### Description

Based on 7 specimens mounted in Hoyer’s medium. Body fusiform, robust, comprising cephalic region, trunk, peduncle and haptor. Body length, including haptor, 530 (370–660; *n* = 7) by 184 (70–230; *n* = 7) width at the level of germarium. Tegument thin, smooth. Cephalic margin broad; cephalic lobes moderately developed; three bilateral pairs of head organs; cephalic glands posterolateral to pharynx. Eyes absent. Accessory granules sometimes scattered in the pharyngeal region. Mouth subterminal. Pharynx spherical, muscular 38 (25–50; *n* = 4) in diameter. Esophagus short. Two intestinal ceca confluent just posteriorly to testis, lacking diverticula. Gonads intercecal, tandem. Germarium 111 (88–133; *n* = 5) long by 53 (43–60; *n* = 5). Vagina not observed; vaginal canal short with aperture sinistroventral. Seminal receptacle rounded. Mehlis’ gland, uterus, oviduct and ootype not observed. Vitellaria extends throughout the trunk, except in areas of other reproductive organs. Testis postgermarial 119 (100–137; *n* = 5) long by 63 (52–80; *n* = 5) wide. Vas deferens looping left intestinal cecum; seminal vesicle a dilatation of vas deferens. Prostatic glands forming a dense mass around of the prostatic reservoir. Prostatic reservoir piriform. Copulatory complex comprising MCO and accessory piece. MCO formed by a sclerotized tube slightly flattened and twisted with the same thickness over its entire length 48 (42–55; *n* = 7) in total length. Accessory piece formed by a single plate of saccular appearance with apparently membranous walls 23 (12–31; *n* = 7); MCO base with a conspicuous flap, 22 (21–23; *n* = 3) long. Peduncle very short. Haptor subhexagonal, with dorsal, ventral anchor/bar complex 117 (60–150; *n* = 7) wide. Hooks with ancyrocephaline distribution. Anchors similar in shape and size, deep and superficial roots well-developed, shaft curved, point straight. Ventral anchor outer 31 (30–32; *n* = 6); inner 19 (17–21; *n* = 6); base 18 (17–20; *n* = 6). Dorsal anchor outer 31 (30–32; *n* = 6); inner 21 (20–22; *n* = 6); base 18 (17–19; *n* = 6). Ventral bar open V-shaped with tapered ends, 60 (54–67; *n* = 6) wide. Dorsal bar V-shaped, with two submedial long projections directed posteriorly, 65 (45–82; *n* = 6). Hooks similar in shape and size, with erect thumb, straight shaft, and point; shank bent back proximally 16 (15–17; *n* = 35). FH loop almost the total shank length.

### Remarks

*Cosmetocleithrum sacciforme* n. sp. differs from all congeneric species mainly in terms of the morphology of the accessory piece, which is formed by a single plate of saccular appearance. The new species is similar to *C. bifurcum* Mendoza-Franco, Mendoza-Palmero & Scholz, 2016 with regard to the presence of a dense mass of prostatic glands in the anterior trunk but differs in the morphology of the anchors and copulatory complex.

## *Cosmetocleithrum basicomplexum* n. sp (Fig. 5)

urn:lsid:zoobank.org:act:EEAF4C09-5CB9-492B-946A-27D665D790B7

**Figure 5:**
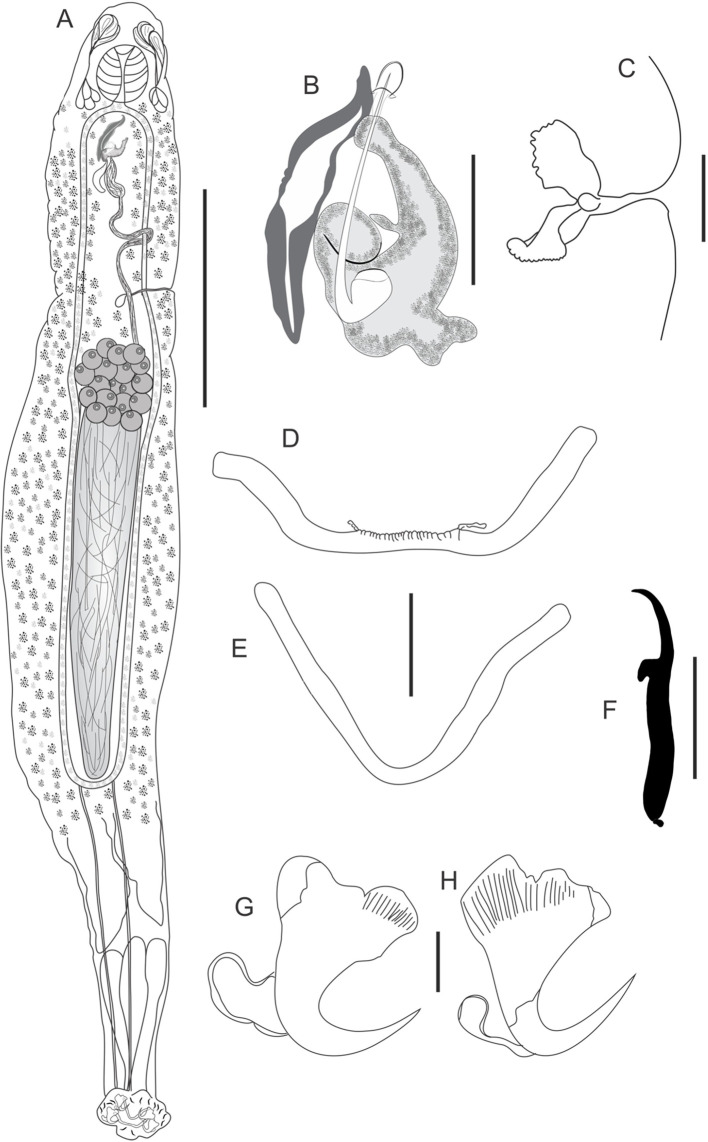
*Cosmetocleithrum basicomplexum* n. sp. from *Oxydoras niger*. (A) Total, ventral view (composite); (B) Copulatory complex, ventral view; C) Vagina; (D) Dorsal bar; (E) Ventral bar; (F) Hook; (G) Dorsal anchor; (H) Ventral anchor. Scale bars: A, 50 μm, B, 40 μm, C, G, H, 20 μm, D, E, 30 μm, F, 10 μm.

Type-host: *Oxydoras niger* (Valenciennes, 1821) (Siluriformes, Doradidae).

Site in host: Gill lamellae.

Type-locality: Juruá River, Acre, Brazil (7°40′34.1″S, 72°39′39.5″W).

Parasitological indexes: Total number of hosts: 7; number of infected hosts: 2; total number of parasites: 30.

Specimens deposited: Holotype CHIOC 40249 a; Paratypes CHIOC 40249 b–q; 40250.

Etymology: The specific name is from Latin (*basis* = base; *complexum* = complex) and refers to the ornamentation that almost surrounds the base of the MCO.

### Description

Based on 23 specimens: 15 mounted in Hoyer’s medium and 8 stained in Gomori’s trichrome. Tegument thin, smooth. Body fusiform, very elongated, comprising cephalic region, trunk, peduncle, and haptor. Body length, including haptor, 2,768 (2,100–3,800; *n* = 23) by 531 (300–1,350; *n* = 23) width at the level of germarium. Cephalic lobes poorly developed. Three bilateral pairs of head organs. Cephalic glands posterolateral to pharynx. One pair of eyes. Mouth subterminal. Pharynx subspherical, muscular 175 (135–250; *n* = 20) by 302 (130–430; *n* = 20) wide. Esophagus short. Two intestinal ceca confluent just posteriorly to testis, lacking diverticula. Gonads intercecal, testes dorsal to germarium. Germarium 196 (180–235; *n* = 9) long by 177 (100–185; *n* = 9). Vagina formed by two chambers; vaginal aperture sinistroventral. Seminal receptacle indistinct. Mehlis’ gland, uterus, oviduct and ootype not observed. Vitellaria extends throughout the trunk, except in areas of other reproductive organs. Testis postgermarial 908 (775–1,025; *n* = 9) long by 152 (125–205; *n* = 9) wide. Vas deferens looping left intestinal cecum; seminal vesicle a dilatation of vas deferens. Prostatic reservoir present. Copulatory complex comprising MCO and accessory piece. MCO a sclerotized coiled tube, with wide base opening, 70 (62–82; *n* = 14) in total length. Straight and robust accessory piece, with an apparently hollow structure and sclerotized walls, serving as a guide for the MCO 76 (62–90; *n* = 14); MCO base broad and sclerotized edges approximately the length of the MCO 77 (50–60; *n* = 14) long. Peduncle very long. Haptor subhexagonal, very small in relation to the body, with dorsal, ventral anchor/bar complex 269 (155–490; *n* = 22) wide. Hooks with ancyrocephaline distribution. Anchors dissimilar in shape, deep and superficial roots well-developed, shaft curved, point straight. Ventral anchor outer 51 (47–55; *n* = 14) long; inner 25 (20–30; *n* = 14); base 37 (32–42; *n* = 14). Dorsal anchor outer 51 (48–55; *n* = 14) long; inner 25 (20–38; *n* = 14); base 35 (32–38; *n* = 14). Ventral bar thin, V-shaped with midline grooves 102 (80–130; *n* = 14) wide. Dorsal bar thin and fragile W-shaped or arch-shaped, tapering in the medial region from where a small and very fragile projection emerges, often almost imperceptible 108 (87–126; *n* = 7) wide. Hooks similar in shape and size, thumb short, depressed, shaft strongly robust through its length, tapered abruptly in the final portion of the shaft, forming a small pointed structure 20 (18–21; *n* = 42). FH loop almost the total shank length.

### Remarks

*Cosmetocleithrum basicomplexum* n. sp is more closely related to *C. confusus*, *C. sacciforme* and *C. ludovicense* regarding the enlarged base of the MCO, but differs from the first two in terms of the morphology of the accessory piece. In the new species, this is straight, with an apparently hollow structure and sclerotized walls opening from half its length, while in *C. confusus* it is a hollow structure with sclerotized walls and truncated termination and in *C. sacciforme* it is sac-shaped with apparently membranous walls. The new species closely resembles *C. ludovicense* n. sp. regarding the morphology of the copulatory complex (*i.e.* MCO and accessory piece) but differs in terms of the shape of anchors and bars. *Cosmetocleithrum basicomplexum* n. sp. shares morphological characteristics with *C. gigas* Morey, Cachique & Babilonia, 2019 regarding the body size and anchor shape but can be differentiated mainly by the morphology of bars. In addition, the hooks of *C. basicomplexum* n. sp. has a shaft that is strongly robust throughout its length, with a small constricted point, as was observed in the hooks of *C. undulatum*.

## *Cosmetocleithrum akuanduba* Soares, Neto and Domingues, 2018 (Figs. 6A–6B)

Type-host: *Hassar gabiru* Birindelli, Fayal & Wosiacki, 2011.

**Figure 6:**
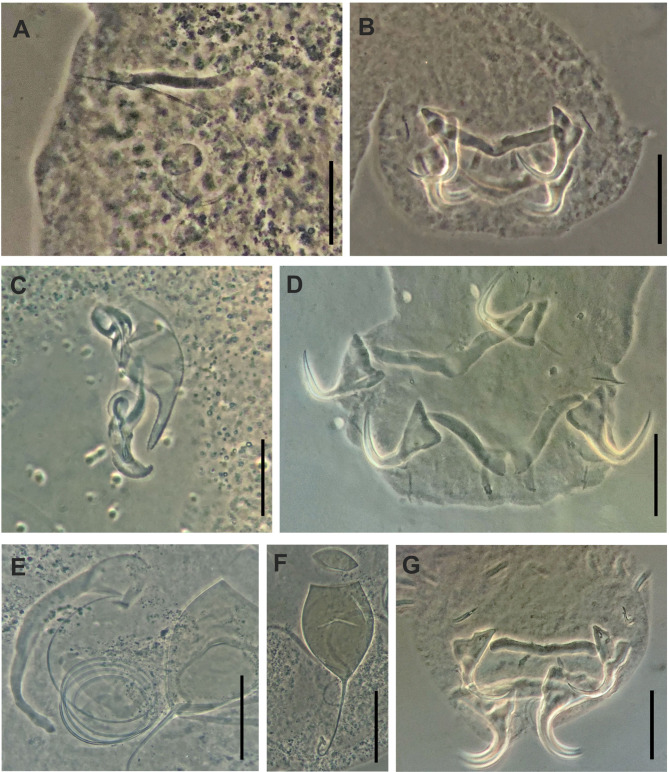
(A, B) *Cosmetocleithrum akuanduba* Soares, Neto and Domingues, 2018: (A) Male copulatory organ; (B) Haptor structures; (C, D) *Cosmetocleithrum confusus* Kritsky, Thatcher and Boeger, 1986: (C) Male copulatory organ; (D) Haptor structures; (E)–(G) *Cosmetocleithrum leandroi* Soares, Neto and Domingues, 2018: (E) Male copulatory organ; (F) Egg; (G) Haptor structures. Scale bars: A, 20 μm, B, D, F, G, 40 μm, C, 30 μm, E, F, 50 μm.

Type-locality: Ilha grande, Xingu River, municipality of Altamira, Pará, Brazil.

Other records: Brazil, *Hassar affinis* (Steindachner, 1881) Marketplace of the Cidade Operária, São Luís, State of Maranhão (2°34′18.0″S 44°11′49.9″W) (present study); *H*. *gabiru* from Iriri River, and Bacajá River, municipality of Altamira, Pará; *H. orestis* (Steindachner, 1875) from Xingu River, Belo Monte Community, municipality of Vitória do Xingu, Pará [21].

Parasitological indexes: Total number of hosts: 1; number of infected hosts: 1; total number of parasites: 15.

### New data

Based on 12 specimens mounted in Hoyer’s medium: Body length, including haptor, 560 (480–650; *n* = 6) by 110 (90–130; *n* = 6) width at the level of germarium. Tegument thin, smooth. Eyes and accessory granules absent. Pharynx spherical, muscular 48 (32–65; *n* = 6) in diameter. Esophagus short. Gonads intercecal, tandem. Testis postgermarial. Copulatory complex comprising MCO and accessory piece. MCO consists of a thin tube forming a semi-ring in a counter-clockwise orientation, J-shaped 94 (79–116; *n* = 12) in total length. Accessory piece formed by a straight rod, presenting a small gutter in the distal portion serving as a guide for the MCO 31 (21–37; *n* = 12) long. Haptor subhexagonal, with dorsal, ventral anchor/bar complex, 90 (70–120; *n* = 9) wide. Anchors similar in shape and size, both of them comprising inconspicuous superficial and deep roots, curved shaft and long and slightly curved point; point acute passing the level of superficial root tip. Ventral anchor with acute superficial root tip 25 (22–29; *n* = 10) inner; 32 (31–34; *n* = 10) outer; base 20 (15–24; *n* = 10) wide; Dorsal anchor with rounded superficial root tip, 26 (23–29; *n* = 10) inner; 32 (30–35; *n* = 10) outer; base 19 (14–22; *n* = 10) wide. Ventral bar slightly arcuate with delicate fracture hatches at medial region and rounded ends 54 (47–63; *n* = 10) wide. Dorsal bar arcuate, V-shaped, with rounded ends and two submedial long projections directed posteriorly 58 (40–66; *n* = 10). Hooks with ancyrocephaline distribution, similar in shape and size; non-dilated shank, 14 (13–15; *n* = 28) long.

### Remarks

According to Soares *et al.* [21], *C. akuanduba* is characterized mainly by the J-shaped MCO; elongated accessory piece with sharp distal region, distal portion with a small gutter and by the heavily sclerotized vagina with short S-shaped vaginal canal. The specimens studied herein were conformable with the original description, with small differences in the size of the MCO and accessory piece, which in the present study were larger than the specimens described by Soares *et al.*: [MCO 94 (79–116); accessory piece 31 (21–37) in the present study *vs* MCO 68 (54–76); accessory piece 23 (18–30)] in Soares *et al.* [21].

## *Cosmetocleithrum confusus* Kritsky, Thatcher & Boeger, 1986 (Figs. 6C–6D)

Type-host: *Oxydoras niger* (Valenciennes), Doradidae.

Type-locality: Janauacá Lake near Manaus, Amazonas, Brazil.

Other records: Brazil, *Oxydoras niger* from Juruá River, State of Acre (7°40′34.1″S, 72°39′39.5″W) (present study); Janauacá Lake, near Manaus, Amazonas State [10]; basin of Solimões River, Amazonas state [20]; Peru, Amazonas River, Iquitos [9].

Parasitological indexes: Total number of hosts: 7; number of infected hosts: 2; total number of parasites: 9.

### New data

Based on 11 specimens mounted in Hoyer’s medium. Body fusiform, elongated, comprising cephalic region, trunk, peduncle, and haptor. Body length, including haptor, 883 (620–1040; *n* = 11) long by 158 (81–185; *n* = 11) wide, width at the level of germarium. Tegument thin, smooth. Copulatory complex comprising MCO and accessory piece. MCO formed by a coiled sclerotized tube, poorly defined coil, 67 (54–93; *n* = 11)) in total length, counter-clockwise orientation. Accessory piece 64 (57–75; *n* = 11) long, non-articulated with MCO, with a proximal portion very tapered and a hollow bulbous portion in the distal region Haptor subhexagonal. Anchors similar in shape; elongate point, short small base; ventral anchor, 31 (31–34; *n* = 10) long, base 23 (20–35; *n* = 10); dorsal anchor, 32 (32–39; *n* = 10), base 24 (22–35; *n* = 10). Ventral bar V-shaped 85 (72–105; *n* = 10) long. Dorsal bar V-shaped, with two submedial long projections directed posteriorly 88 (105–87; *n* = 10) long. Hooks similar in shape and size, tapered shaft and point thumb, straight depressed thumb, slender shank 16 (13–17; *n* = 40).

### Remarks

The morphology of *C. confusus* from the present study is in agreement with the original description of Kritsky *et al.* [10], differing only in relation to total body length and bars length, which in the present study were greater than in the specimens described by Kritsky *et al.*: body length 883 (620–1,040) long by 158 (81–185) wide, ventral bar 85 (72–105) long and dorsal bar 88 (105–87) long, in the present material *vs* body length 564 (449–706) long by 158 (81–185) wide, dorsal bar 53 (47–62) long and ventral bar 59 (47–74) long in the material of Kritsky *et al.* [10].

## *Cosmetocleithrum leandroi* Soares, Neto & Domingues, 2018 (Figs. 6E–6F)

Type-host: *Hassar gabiru* Birindelli, Fayal & Wosiacki, 2011, Doradidae.

Type-locality: Bacajá River, municipality of Altamira, Pará, Brazil.

Others records: Brazil, *Hassar affinis* (Steindachner, 1881) from Market-place of the Cidade Operária, São Luís, State of Maranhão (2°34′18.0″S 44°11′49.9″W) (present study); *H. gabiru* from Ilha Grande, Xingu River, and Iriri River, municipality of Altamira, Pará [21].

Parasitological indexes: Total number of host: 1; number of infected hosts: 1; total number of parasites: 93.

### New data

Based on 13 specimens mounted in Hoyer’s medium. Body fusiform, elongated, comprising cephalic region, trunk, peduncle, and haptor. Body length, including haptor, 933 (650–1,250; *n* = 13) long by 119 (110–175; *n* = 13) wide width at the level of germarium. Tegument thin, smooth. Eyes and accessory granules absent. Pharynx spherical, muscular 62 (50–80; *n* = 8) in diameter. Vagina heavily sclerotized, vaginal pore sinistroventral; vaginal canal very long, convoluted, looping towards the MCO. Copulatory complex comprising MCO and accessory piece. MCO formed by a coiled sclerotized tube with a bulbous structure in the distal region 612 (546–664; *n* = 7) in total length, with 3½ to 4½ counter-clockwise rings. Accessory piece 112 (98–138; *n* = 13) long, not articulated with MCO, comprising a sigmoid rod, with cup-shaped distal region. Peduncle long. Haptor subhexagonal, 109 (110–175; *n* = 10) wide. Ventral anchors with pointed superficial root and deep root broad, slightly curved shaft and point, outer 47 (44–50; *n* = 13) long, inner 34 (31–39; *n* = 13), base 30 (24–33; *n* = 13); dorsal anchor, with pointed superficial root and deep root short, slightly curved shaft and point, outer 37 (31–43; *n* = 13), inner 33 (29–36; *n* = 13), base 23 (20–30; *n* = 13). Ventral bar straight with knobbed ends 49 (42–55; *n* = 13) long, by 6 (5–10; *n* = 13) wide. Dorsal bar straight, with two submedial long projections directed posteriorly 55 (50–65; *n* = 13) long by 7 (5–8; *n* = 13). Hooks similar in shape and size, inconspicuous rounded thumb, shaft straight, short, slightly dilated 15 (14–15; *n* = 40). Egg oval, with filament opposite to opercular end, 84 (80–87; *n* = 5) long by 50 (47–52; *n* = 5) wide; filamentous 58 (47–70; *n* = 5).

### Remarks

*Cosmetocleithrum leandroi* was described from the gill filaments of *Hassar gabiru*, in the state of Pará, Brazil. According to the original description, *C. leandroi* is characterized by a MCO comprising a coil of about 3½ rings, a sigmoid accessory piece with a cup-shaped distal portion, a single type of hooks, and anchors with poorly differentiated roots. The specimens studied here, based on newly collected specimens from *H. affinis*, were in accordance with the original description, while presenting some differences in morphology and measurements, compared with the original material, deposited in CHIOC (Holotype 39053 a, paratypes 39053 b–f, vouchers 39054 a–c): body length 933 (650–1,250) long by 119 (110–175) wide, MCO with 3½ to 4½ counter-clockwise rings, ventral and dorsal anchors with pointed superficial root and deep root developed and hooks with slightly dilated shank, in the present material *vs* body length 712 (575–835) long by 132 (102–157) wide, MCO with 3½ counter-clockwise rings, superficial and deep roots poorly developed and hooks with non-dilated shank in the original description and type material examined.

## Discussion

*Cosmetocleithrum* is one of the most diverse genera of dactylogyrids parasitizing neotropical catfishes [4]. It is characterized by the presence of two submedial ribbon-like projections on the dorsal bar, a copulatory complex comprising a variably coiled cirrus with counter-clockwise rings and an elaborate accessory piece non-articulated to the cirrus base [10].

So far, species of *Cosmetocleithrum* have been found in fishes caught in Argentina (1), Brazil (18), and Peru (4). Among these, 14 species parasitize hosts of the Doradidae: *C. akuanduba*, *C. bifurcum*, and *C. leandroi* parasitizing *Hassar gabiru*; *Cosmetocleithrum phryctophallus* Soares, Santos Neto & Domingues, 2018 parasitizing *Hassar orestis* (Steindachner); *C. confusus*, *Cosmetocleithrum gigas*, *C. gussevi* Kritsky, Thatcher & Boeger, 1986, *Cosmetocleithrum parvum* Kritsky, Thatcher & Boeger, 1986, *Cosmetocleithrum rarum* Kritsky, Thatcher & Boeger, 1986 and *Cosmetocleithrum sobrinus* Kritsky, Thatcher & Boeger, 1986 parasitizing *O. niger*; *Cosmetocleithrum tortum* Mendoza-Franco, Mendoza-Palmero & Scholz, 2016 parasitizing *Nemadoras hemipeltis* (Eigenmann), *Cosmetocleithrum infinitum* Morey, Rojas & Cachique, 2022 parasitizing *Anadoras grypus* (Cope); *C. falsunilatum* parasitizing *Megalodoras uranoscopus* (Eigenmann & Eigenmann) and *Cosmetocleithrum trachydorasi* (Acosta, Scholz, Blasco-Costa, Alves, Silva & 2018) Cohen, Justo, Gen & Boeger, 2020 parasitizing *Trachydoras paraguayensis* (Eigenmann & Ward).

Cohen *et al.* [4] proposed two morphologically distinguished groups among the species of *Cosmetocleithrum*: (1) species that resemble the type species, *Cosmetocleithrum gussevi*, which present non-articulated bars and a distally bifid accessory piece, often resembling a hook (*C. parvum*, *C. rarum*, *C. sobrinus*, *Cosmetocleithrum longivaginatum* Suriano & Incorvaia, 1995, *C. striatuli*, *C. laciniatum*, *C. phryctophallus* and *C. gigas*); and (2) species with articulated bars and a variably shaped accessory piece (*e.g. C. confusus*, *C. bulbocirrus*, *C. tortum* and *C. bifurcum*). Subsequently, new species of *Cosmetocleithrum* were proposed and can be placed in those groups. Among these, *C. berecae* Cohen, Justo, Gen & Boeger, 2020, *C. nunani* Cohen, Justo, Gen & Boeger, 2020, *C. falsunilatum* Feronato, Razzolini, Morey & Boeger, 2022, *C. galeatum* Yamada, Yamada & Silva, 2020, *C. spathulatum* Yamada, Yamada & Silva, 2020 and *C. baculum* Yamada, Yamada & Silva, 2020 were placed in group 1. In the description of *Cosmetocleithrum infinitum*, Morey *et al.* [13] present images that showed the articulation on the ventral bar. Among the new species proposed in the present study, only *C. ludovicense* and *C. sacciforme* present articulated bars and thus are included in group 2, while *C. undulatum*, *C. brachylecis* and *C. basicomplexum* are included in the group 1.

Nevertheless, in the phylogenetic analysis of *Cosmetocleithrum* conducted by Mendoza-Palmero *et al.* [11], the phylogenetic position of the species analyzed corresponded partially to the morphological categories proposed by Cohen *et al.* [4]. Their study [11] provides suggestions for further studies regarding *Cosmetocleithrum* spp., including the molecular characterization of the remaining species of this genus in order to evaluate the phylogenetic positions of all the species.

Mendoza-Palmero *et al.* [11] stated that more than 15 species of *Cosmetocleithrum* have been described over recent years, thereby adding new morphological characteristics that can be included in the diagnosis of the genus. They cited *C. bifurcum* (a member of the “doradid group”) and *C. baculum* (a member of the “auchenipterid group”), which have dissimilar hooks, but *C. nunani* (also a member of the auchenipterid group) presents two morphologically distinct hooks. Regarding the species described in the present paper, *C. undulatum* (a member of the doradid group) can be included in this set of species with two morphological distinct hooks, thus showing that it is not related to the host family. Considering the position of the vagina, a feature also mentioned by Mendoza-Palmero *et al.* [11], the species described herein are concordant with almost all species of the genus, presenting a sinistral aperture, with the exception of *C. tortum*.

Despite the high diversity of catfish in the Amazon region and the economic importance of some of these species, knowledge of the helminth fauna parasitizing these fish is still fragmentary and far from sufficient [12]. Moreover, no studies on the helminth fauna of many of these fish have yet been conducted, as is the case of *P. brachylecis*, which is a thorny catfish endemic to the basins of the Mearim River, Pindaré River, Itapecuru River and Parnaíba River, in northeastern Brazil [16]. The new species described in the present study and the new records of hosts for *P. brachylecis* demonstrate that there is a real need to expand such studies, especially with regard to endemic fish species and those that have recently been described. The finding, more than 30 years later, of two new species in *O. niger*, the type host of the first four species that were proposed for *Cosmetocleithrum*, also demonstrates that studies on these hosts are necessary and should take into consideration the ecological processes related to the host-parasite association.
